# Association of healthy lifestyle behaviors with incident gastroesophageal reflux disease in a large population-based prospective cohort

**DOI:** 10.1016/j.pmedr.2025.103276

**Published:** 2025-10-24

**Authors:** Zhiyu Wang, Siwei Lin, Hanwen Liu, Jianlong Zhou, Enpei Tang, Zhihui Xi, Jiabin Zheng, Wei Yang

**Affiliations:** a Medical Research Institute, Guangdong Provincial People's Hospital (Guangdong Academy of Medical Sciences), Southern Medical University, Guangzhou 510080, China.; b Guangdong Province Key Laboratory of Molecular Tumor Pathology, Department of Pathology, School of Basic Medical Sciences, Southern Medical University, Guangzhou 510515, China.; c Department of Gastrointestinal Surgery, Guangdong Provincial People's Hospital (Guangdong Academy of Medical Sciences), Southern Medical University, Guangzhou, Guangdong 510080, China; d School of Medicine, South China University of Technology, Guangzhou, Guangdong 510006, China; e Department of Neurosurgery, Ganzhou Hospital of Guangdong Provincial People's Hospital, Ganzhou Municipal Hospital, Ganzhou 341000, China

**Keywords:** Gastroesophageal reflux disease, Healthy lifestyle behaviors, Prospective cohort study, UK biobank, Prevention strategies

## Abstract

**Objective:**

Examine the association between healthy lifestyle behaviors (smoking, optimal sleep, high-intensity physical activity, and moderate alcohol consumption) and the risk of Gastroesophageal reflux disease (GERD).

**Methods:**

We conducted a prospective cohort study of UK Biobank. Four healthy lifestyle behaviors were assessed at baseline. Participants were followed for 14.2 years until September 15, 2024, and GERD incidence was recorded. Multi variable-adjusted Cox models estimated HRs and 95 % CIs for the association between healthy behaviors and GERD risk.

**Results:**

Among 108,239 participants, 8143 (7.5 %) developed GERD during follow-up. Distribution of healthy lifestyle behaviors was: 14.5 % reported none, 38.6 % reported one, 34.1 % reported two, and 12.7 % reported three to four behaviors. Participants with more behaviors had progressively lower GERD risks, with HRs of 0.92 (95 % CI 0.86, 0.98), 0.82 (0.77, 0.87), and 0.68 (0.63, 0.75), respectively (P for trend <0.01). Never smoking (HR: 0.84; 95 % CI: 0.81, 0.88), high physical activity (0.86; 0.83, 0.90), and optimal sleep (0.70; 0.65, 0.74) were independently associated with reduced GERD risk.

**Conclusions:**

Adopting multiple healthy lifestyle behaviors significantly reduces GERD risk, emphasizing the importance of lifestyle modifications in GERD prevention.

## Highlights

• Healthy behaviors reduce Gastroesophageal reflux disease risk.
• Each additional healthy behavior lowers Gastroesophageal reflux disease risk.
• Never smoking, high physical activity, and optimal sleep independently reduce the risk.
• Depressed people need ≥ 3 healthy behaviors to reduce the risk.
• Lifestyle changes are key for Gastroesophageal reflux disease prevention.

## 1 Introduction

Gastroesophageal reflux disease (GERD), a chronic condition affecting the upper gastrointestinal tract(Nwokediuko, 2012), has seen a rising incidence worldwide, posing a significant public health challenge(Nirwan et al., 2020). Characterized primarily by heartburn and regurgitation, GERD is one of the most common complaints in clinical practice, affecting approximately 10–20 % of adults in Western countries and an increasing number of individuals in Asia and other regions. The condition cause significantly impairs patients' health-related quality of life, leading to sleep disturbances, reduced productivity, and diminished overall well-being(Ofman, 2003). Management of GERD often necessitates a combination of lifestyle modifications, long-term pharmacological treatment (e.g., proton pump inhibitors, PPIs), and, surgical interventions (Lee and McColl, 2013).

The pathogenesis of GERD is multifactorial, involving a complex interplay of anatomical, physiological, and lifestyle-related factors. Key mechanisms include increased compliance at the esophagogastric junction (OGJ), which allows for abnormal reflux of gastric contents into the esophagus, and an elevated pressure gradient across the OGJ, often exacerbated by conditions such as obesity or hiatal hernia(Miller et al., 2011). Additionally, abnormalities in meal distribution can contribute to reflux episodes. Beyond these mechanical factors, emerging evidence suggests that genetic predisposition, visceral hypersensitivity, and alterations in esophageal motility may also play significant roles in the disease's etiology(Orlando, 2001).

Current treatment strategies for GERD primarily focus on symptom management through pharmacological interventions, which reduce gastric acid production, or surgical procedures aimed at reinforcing the anti-reflux barrier. While these approaches are effective in alleviating symptoms for many patients, they are not without limitations. Long-term use of PPIs has been associated with adverse effects, including an increased risk of infections, nutrient deficiencies, and bone fractures. Surgical interventions, though beneficial for some, carry risks of complications and may not be suitable for all patients(Zhang et al., 2021). Moreover, these treatments are typically initiated after the disease has already manifested, often resulting in significant economic burdens and a diminished quality of life for patients. Consequently, there is a growing recognition of the need to shift focus toward primary prevention, aiming to address modifiable risk factors before the onset of GERD(Maret-Ouda et al., 2020).

Dietary and lifestyle modifications are commonly recommended as first-line interventions to mitigate GERD symptoms and prevent disease progression. For instance, weight loss, avoidance of trigger foods, and elevation of the head during sleep are frequently advised. However, despite their widespread recommendation, there is limited prospective evidence to support the efficacy of these measures in preventing GERD. Furthermore, the combined impact of multiple healthy lifestyle behaviors on GERD risk has not been thoroughly investigated(Sethi and Richter, 2017). To address this gap, we conducted a large-scale prospective study utilizing data from the UK Biobank.

Previous studies have identified several modifiable lifestyle factors that are independently associated with GERD risk. These include smoking, which has been shown to impair esophageal motility and reduce salivary bicarbonate secretion(Ness-Jensen et al., 2016); poor sleep quality, which may exacerbate nocturnal reflux episodes(Ma et al., 2024); physical inactivity, which is linked to obesity and increased intra-abdominal pressure(Taraszewska, 2021); and excessive alcohol consumption, which can increase acid production(Ness-Jensen and Lagergren, 2017). While the individual effects of these factors have been explored, their combined impact on GERD risk remains poorly understood. We hypothesized that adopting a combination of healthy lifestyle behaviors—such as smoking abstinence, optimal sleep patterns, regular physical activity, and moderate alcohol consumption—could collectively reduce the incidence of GERD. By leveraging the UK Biobank cohort, we aimed to evaluate this hypothesis and provide robust evidence to inform preventive strategies.

In this study, we assessed the association between these four key lifestyle factors and the incidence of GERD, adjusting for potential confounders such as age, sex, body mass index (BMI), and comorbidities. Our findings have important implications for both clinical practice and public health, offering insights into how lifestyle modifications can reduce the burden of GERD. By identifying specific behaviors that contribute to GERD prevention, we hope to empower individuals to make informed choices and guide healthcare providers in delivering targeted interventions. Ultimately, this research highlights the potential of lifestyle medicine as a cornerstone of GERD prevention to improve patient outcomes and reduce healthcare costs.

## 2 Methods

### 2.1 Study setting and participant selection

The UK Biobank is a large-scale prospective cohort study that recruited 502,170 participants aged 37 to 73 years between 2006 and 2010. Participants were invited via mail and completed baseline assessments, including touch-screen questionnaires on demographics, health, and lifestyle, oral interviews, physical examinations, and biological sample collection at one of 22 assessment centers across the UK. Follow-up procedures were implemented to monitor and update participants' health status. A detailed description of the UK Biobank study design is available in the literature(Sudlow et al., 2015).

**Fig. 1:**
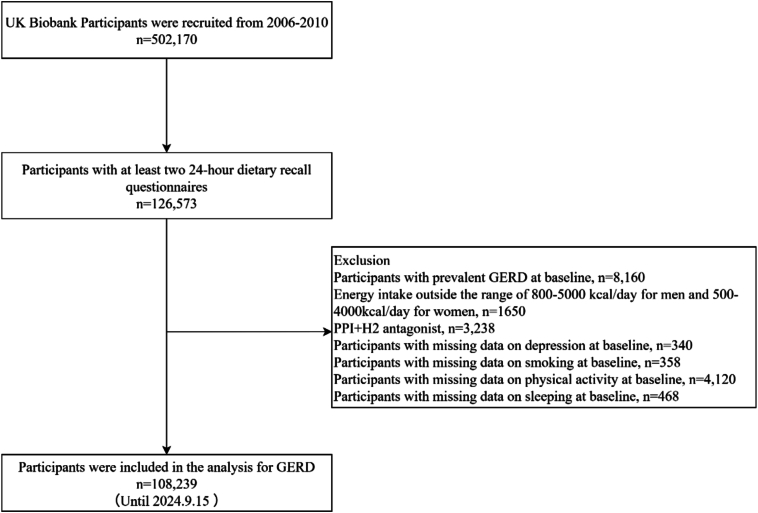
Flow chart of eligible UK Biobank participants (2006–2024). GERD, Gastroesophageal reflux disease; PPIs, Proton pump inhibitors.

To assess dietary habits, we restricted our analysis to participants who completed at least two 24-h dietary recall questionnaires (*n* = 126,573)(De Boer et al., 2011), as prior studies have demonstrated the reproducibility of this method(Carter et al., 2019). To minimize inference bias, we excluded participants with unrealistic energy intakes (men: < 800 or > 5000 kcal/day; women: < 500 or > 4000 kcal/day, *n* = 1650)(Yévenes-Briones et al., 2022), and those with prevalent GERD at baseline (*n* = 8160). Participants who were missing data on any of the four lifestyle behaviors (physical activity, *n* = 4120; smoking, *n* = 358; sleeping, *n* = 468) or key covariates (used proton pump inhibitors (PPIs), *n* = 3238) and participants with depression at baseline (*n* = 340), were excluded to reduce the probability of inferential bias. And the analysis included only those participants whose exposure, outcome, and key covariates were all recorded in UK Biobank. After exclusions, 108,239 participants were included in the final analysis (Fig.1).

### 2.2 Exposure assessment

We evaluated four healthy lifestyle behaviors: never smoking, optimal sleep, high-intensity physical activity, and moderate alcohol consumption. Participants were categorized based on the number of healthy behaviors they exhibited (zero, one, two, or three to four). Due to the limited number of participants with three (*n* = 12,403) or four (*n* = 1383) healthy behaviors, these groups were combined for analysis. All lifestyle behaviors were assessed using structured, self-reported questionnaires. (**Text.S1**).

### 2.3 Outcome determination

The primary outcome was the incidence of GERD, defined by the International Classification of Diseases, 10th revision (ICD-10) code K21. GERD cases were identified using data from primary care records, hospital inpatient records, death registries, and self-reported medical conditions. The date of the first GERD diagnosis was recorded for each case. (**Text.S1**).

### 2.4 Covariate identification

Covariates related to healthy lifestyle behaviors and GERD were identified through a comprehensive review of observational studies, systematic reviews, and consensus reports. These included sociodemographic factors (age, sex, BMI) and medical factors (depression). (**Text.S1**) Sociodemographic data were collected via touch-screen questionnaires at baseline, while medical conditions were verified using primary care data, inpatient records, death registries, and self-reported information. Further details on data collection and linkage are available on the UK Biobank website (www.ukbiobank.ac.uk).

### 2.5 Patient and public engagement

The UK Biobank study design incorporated extensive public input during its development. However, patients were not involved in formulating the research questions, outcome measures, study design, or implementation. Additionally, patients were not consulted for input on the interpretation or presentation of results. The study met the institution's or the data curator's guidelines for protection of human subjects concerning safety and privacy.

### 2.6 Ethics approval

This study obtained ethical approval from the North West Multi-Center Research Ethics Committee, the National Information Governance Board for Health and Social Care in England and Wales, and the Community Health Index Advisory Group in Scotland.

### 2.7 Statistical analyses

The baseline characteristics were utilized to describe the distribution of healthy lifestyle behaviors. Categorical variables were presented as numbers (percentages), and comparisons between groups were performed using chi-square tests or Fisher's exact tests, as applicable. Continuous variables were presented as the mean (standard deviations), and comparisons between groups were carried out using analysis of variance tests or Welch tests, depending on the appropriateness of the data.

At the time of analysis, the data from the UK Biobank were current up to September 15, 2024, which was designated as the end of the study. Person-years of follow-up were computed commencing from the first interview at baseline until the occurrence of GERD, death, loss of follow-up, or the end of the study, whichever transpired first.

Cox proportional hazards models were used to estimate HRs and 95 % CIs for the association between healthy lifestyle behaviors and GERD incidence. Covariates adjusted for in the models included age, sex, depression, and BMI. The proportional hazards assumption was tested using Schoenfeld residuals, with no significant violations detected.

We also conducted several sensitivity analyses: we first adopted an alternative definition of healthy alcohol drinking, where moderate intake was no longer considered healthy and only abstainers were classified as having healthy alcohol behaviors; second, we excluded participants diagnosed with GERD within the first two years of follow-up; third, we included educational background and ethnicity as confounding factors in the analysis; and finally, to explore the independent impact of each of the four lifestyle behaviors, we conducted separate analyses for each while adjusting for other behavioral factors in the model.

All analyses were performed using IBM SPSS Statistics version 26.0 (IBM Corporation, Armonk, NY, USA). Two-sided tests were used, and a *P*-value <0.05 was considered statistically significant.

## 3 Results

### 3.1 Baseline characteristics

Table 1 offers a detailed exposition of the baseline characteristics of the study participants, which were methodically stratified based on the count of healthy lifestyle behaviors. The study cohort comprised a total of 108,239 individuals, with an average age of 56.3 years and a female constituency accounting for 55.5 % of the sample. Notably, 15,714 participants, constituting 14.5 % of the total, did not exhibit any of the four healthy lifestyle behaviors under investigation. In sharp contrast, 41,784 individuals (38.6 %) demonstrated one such behavior, 36,955 (34.1 %) adhered to two behaviors, and 13,786 (12.7 %) maintained three to four healthy lifestyle behaviors. When juxtaposed with those lacking all four healthy lifestyle behaviors, participants who adhered to three to four of these behaviors were preponderantly of a younger age demographic, were more likely to be female, and boasted a relatively lower BMI. Additionally, they manifested a decreased prevalence of depression.

**Table 1:** Baseline characteristics of the adult participants, UK Biobank (2006–2024), by the number of healthy lifestyle behaviors.

		Number of healthy lifestyle behaviors a				
						
Variables	Total (*n* = 108,239)	0 (*n* = 15,714)	1 (*n* = 41,784)	2 (*n* = 36,955)	3–4 (*n* = 13,786)	*P*⁎
n (%)/mean (SD)						
Age	56.3 (7.9)	57.6 (7.6)	56.7 (7.8)	55.9 (7.9)	55.0 (8.1)	<0.01
BMI	26.5 (4.5)	27.6 (4.9)	26.8 (4.6)	26.1 (4.2)	25.6 (3.9)	<0.01
Sex						<0.01
Female	60,097 (55.5)	8273 (52.7)	23,551 (56.4)	20,806 (56.3)	7467 (54.2)	
Male	48,142 (44.5)	7441 (47.4)	18,233 (43.6)	16,149 (43.7)	6319 (45.8)	
Depression						<0.01
No	97,677 (90.2)	13,619 (86.7)	37,327 (89.3)	33,828 (91.5)	12,903 (93.6)	
Yes	10,562 (9.8)	2095 (13.3)	4457 (10.7)	3127 (8.5)	883 (6.4)	
Never smoking						<0.01
No	45,452 (42.0)	15,714 (100.0)	21,574 (51.6)	7436 (20.1)	728 (5.3)	
Yes	62,787 (58.0)	0 (0.0)	20,210 (48.4)	29,519 (79.9)	13,058 (94.7)	
Optimal sleep						<0.01
No	90,013 (83.2)	15,714 (100.0)	39,143 (93.7)	29,287 (79.3)	5869 (42.6)	
Yes	18,226 (16.8)	0 (0.0)	2641 (6.3)	7668 (20.8)	7917 (57.4)	
High level of vigorous physical activity						<0.01
No	53,819 (49.7)	15,714 (100.0)	26,468 (63.3)	10,468 (28.3)	1169 (8.5)	
Yes	54,420 (50.3)	0 (0.0)	15,316 (36.7)	26,487 (71.7)	12,617 (91.5)	
Moderate alcohol intake						<0.01
No	85,237 (78.8)	15,714 (100.0)	38,167 (91.3)	26,719 (72.3)	4637 (33.6)	
Yes	23,002 (21.3)	0 (0.0)	3617 (8.7)	10,236 (27.7)	9149 (66.4)	

BMI, body mass index; SD, standard deviation.

⁎ The p-value for continuous variables was calculated using the Mann Whitney *U* test, while the *p*-value for categorical variables was calculated using the chi-square test.

a Healthy lifestyle behaviors included never smoking, a high level of vigorous physical activity (in the highest 50 % of the cohort), moderate alcohol intake (5–15 g/day) and optimal sleep (having a sleep duration of between 7 and 9 h/day, finding it fairly easy or very easy to get up in the morning and never or rarely having insomnia and narcolepsy).

### 3.2 Healthy lifestyle behaviors and GERD

Over a mean follow-up period of 14.2 years, a cumulative total of 8143 cases (7.5 %) of GERD were documented. As illustrated in Table 2, subsequent to making adjustments for age (< 50 years old, 50–59 years old, or ≥ 60 years old), sex (female or male), BMI (< 25 or ≥ 25), and depression (yes or no), it was conclusively determined that a greater degree of adherence to healthy lifestyle behaviors was strikingly correlated with a mitigated risk of developing GERD (all *P* < 0.05). In relation to the absence of all four healthy lifestyle behaviors, the adjusted hazard ratios corresponding to the adherence of one, two, or three to four behaviors were 0.92 (0.86, 0.98), 0.82 (0.77, 0.87), and 0.68 (0.63, 0.75), respectively (*P* < 0.01). As graphically represented in **Fig.S1**, the survival-risk curves provided further substantiation that an augmented number of healthy behaviors was concomitant with a diminished exposure risk to GERD. The probability of developing GERD over time in people with three to four healthy lifestyles is much lower than that in people without healthy lifestyles.

**Table 2:** Cox proportional hazards models investigating the associations between adult participants' healthy lifestyle behaviors and the risk of incident Gastroesophageal reflux disease (GERD) from UK Biobank (2006–2024) ⁎

	Number of healthy lifestyle behaviors a				
	0	1	2	3–4	*P* value for trend
No. of participants	15,714 (14.5)	41,784 (38.6)	36,955 (34.1)	13,786 (12.7)	
Person-years	89,500.0	224,666.7	151,235.3	51,333.3	
No. of GERD events	1432 (9.1)	3370 (8.1)	2571 (7.0)	770 (5.6)	
Adjusted hazard ratio b (95 %CI)	1.00(reference)	0.92 (0.86, 0.98)	0.82 (0.77, 0.87)	0.68 (0.63, 0.75)	<0.01

GERD, Gastroesophageal reflux disease.

⁎ Values are numbers (percentages) unless stated otherwise.

a Healthy lifestyle behaviors included never smoking, a high level of vigorous physical activity (in the highest 50 % of the cohort), moderate alcohol intake (5–15 g/day) and optimal sleep (having a sleep duration of between 7 and 9 h/day, finding it fairly easy or very easy to get up in the morning and never or rarely having insomnia and narcolepsy).

b Hazard ratios were adjusted for age (< 50, 50–59 or ≥ 60 years), sex (female or male), BMI (< 25 or ≥ 25) and depression (yes or no).

### 3.3 Sensitivity and separate analyses

**Fig. 2:**
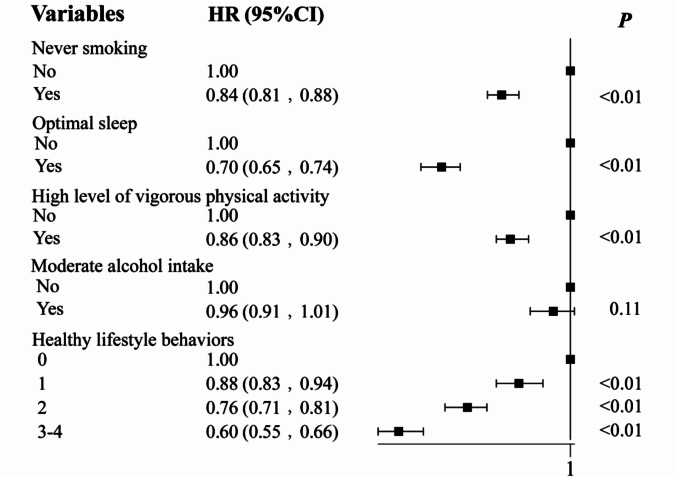
**Cox proportional hazards models investigating the associations of each adult participants' healthy lifestyle behavior with the risk of Gastroesophageal reflux disease from UK Biobank (2006–2024).** Healthy lifestyle behaviors included never smoking, a high level of vigorous physical activity (in the highest 50 % of the cohort), moderate alcohol intake (5–15 g/day) and optimal sleep (having a sleep duration between 7 and 9 h/day, finding it fairly easy or very easy to get up in the morning, and never or rarely having insomnia and narcolepsy). Hazard ratios were adjusted for age (< 50, 50–59 or ≥ 60 years), sex (female or male), BMI (< 25 or ≥ 25) and depression (yes or no). The healthy lifestyle behaviors were also adjusted for each other: never smoking (yes or no), optimal sleep (yes or no), a high level of vigorous physical activity (yes or no)，and moderate alcohol (yes or no); ‘no’ was the reference group for each behavior. For estimates of the association between adhering to 3 to 4 healthy lifestyle behaviors, the reference group comprised those who did not perform any of the behaviors.

Sensitivity analysis using zero alcohol consumption as the reference for healthy drinking behavior (**Table S1**) yielded estimates consistent with the primary analysis presented in Table 2. Sensitivity analysis was conducted by excluding cases that occurred in the first two years of follow-up to mitigate potential reverse causation (**Table S2**). Sensitivity analysis was conducted on educational attainment and race as confounding factors, and the results were similar to those of the previous ones (**Table S3**). Finally, in the discrete examinations of the four lifestyle behaviors, the practice of never smoking (0.84; 0.81, 0.88), a high level of vigorous physical activity (0.86; 0.83, 0.90), and optimal sleep (0.70; 0.65, 0.74) were each significantly and independently inversely related to the incidence of GERD (*P* < 0.01), albeit with a relatively lesser magnitude of association than that observed for adhering to three to four behaviors. While no pronounced independent association was detected for moderate alcohol consumption, the effect size was in close proximity to attaining statistical significance (Fig.2).

## 4 Discussion

In this study, our comprehensive analyses demonstrated that individuals with a combination of healthy lifestyle behaviors—including smoking abstinence, optimal sleep, high-intensity physical activity, and moderate alcohol consumption—were strongly associated with a low incidence of GERD. Notably, we observed that individuals with depressive symptoms who adhered to only one or two healthy lifestyle behaviors did not experience a significant reduction in GERD prevalence. To effectively lower GERD risk in this population, it is recommended to adopt three or more healthy lifestyle behaviors. These findings highlight the importance of a holistic approach to lifestyle modification, particularly for individuals with co-morbid mental health conditions, who may require more intensive interventions to achieve meaningful health benefits.

To our knowledge, this is one of the first prospective cohort studies to establish a relationship between a combination of healthy lifestyle behaviors and a low incidence of GERD. Our findings align with a recent cross-sectional study of 116,671 US women, which also found that a healthy lifestyle was associated with a lower risk of GERD(Mehta et al., 2021). The consistency of these results across different study designs and populations further strengthens the evidence supporting the role of lifestyle factors in GERD prevention. Moreover, our study extends previous findings by quantifying the cumulative effect of multiple healthy behaviors, providing a more nuanced understanding of their combined impact on GERD risk.

Although lifestyle modifications are often recommended for managing GERD symptoms, their role in preventing the disease has been understudied(Commisso and Lim, 2019). GERD is a multifactorial condition influenced by biological, genetic, psycho-social, and environmental factors. Our findings underscore the importance of lifestyle adjustments in the primary prevention of GERD, suggesting that healthy lifestyle choices can mitigate the impact of these etiological factors(Richter and Rubenstein, 2018). For instance, smoking abstinence may reduce esophageal mucosal damage, while optimal sleep and physical activity may improve esophageal motility and reduce intra-abdominal pressure, thereby lowering GERD risk. These mechanisms, though not fully elucidated, provide a plausible biological basis for our observations.

Primary health care providers, as the first point of contact for patients, play a critical role in implementing interventions to modify health behaviors and prevent GERD(Keyworth et al., 2020; Sandhu and Fass, 2017). Routine consultations offer an opportunity to educate patients about the importance of healthy lifestyle choices and provide personalized recommendations. For example, clinicians can encourage smoking cessation, promote regular physical activity, and offer guidance on improving sleep hygiene. Additionally, support from national and local authorities is essential to create an environment that promotes healthy lifestyle behaviors(Ho et al., 2024). Public health campaigns, policy changes (e.g., smoking bans, subsidies for gym memberships), and community-based programs can complement individual-level interventions, fostering a culture of health and wellness. Multilevel efforts are needed to encourage the adoption of these four recommended behaviors for GERD prevention in the general population.

Previous studies have explored the individual effects of these lifestyle behaviors on GERD risk. Our study confirmed that smoking abstinence is closely associated with a reduced incidence of GERD. While existing research suggests that smoking delays gastric emptying(Miller et al., 1989), this hypothesis requires further mechanistic investigation(Scott et al., 1992). Additionally, prospective cohort studies have highlighted the role of the gut-brain axis in GERD pathogenesis, indicating a bidirectional relationship between mental health and GERD(Gong et al., 2023). Given the higher prevalence of smoking among individuals with mental disorders(Lasser et al., 2000) and the association between anxiety/depression and smoking behavior(Dome et al., 2010), smoking may serve as a mediator between mental health and GERD risk. Further research is needed to explore this potential mechanism, as it could inform targeted interventions for high-risk populations.

Optimal sleep was also significantly associated with a lower incidence of GERD. Sleep is regulated by circadian rhythms, which directly influence gastrointestinal function(Orr et al., 2020). Disruptions in circadian rhythms may increase GERD risk by altering esophageal motility and increasing nocturnal acid exposure, while severe reflux can exacerbate sleep disorders(Elkalawy et al., 2024). Thus, improving sleep quality is crucial for GERD prevention. Interventions such as cognitive-behavioral therapy for insomnia and lifestyle modifications (e.g., avoiding late-night meals) may be particularly effective in breaking the vicious cycle between poor sleep and GERD.

We found that high-intensity physical activity was inversely related to GERD incidence. A meta-analysis suggested that maintaining high physical activity levels reduces GERD risk, particularly among older adults and smokers(Yu et al., 2024). Achieving the recommended 150 min of weekly physical activity may significantly lower GERD prevalence, consistent with our findings. Additionally, physical activity has been shown to reduce inflammatory markers, potentially mitigating age-related pro-inflammatory diseases and GERD risk(Hamer et al., 2012). These anti-inflammatory effects, combined with improvements in body weight and gastrointestinal motility, may explain the protective role of physical activity against GERD.

While alcohol consumption can affect gastrointestinal motility and permeability(Bode and Bode, 1997), our study found that moderate alcohol consumption was not significantly associated with GERD incidence. Among the four behaviors, moderate alcohol consumption had the weakest individual association with GERD prevention, but its combined effect with the other behaviors was notable (Pan et al., 2019). We also analyzed the relationship between non-alcohol consumption and gastroesophageal reflux and found that the results between the two alone were not significant either.

Although our research results are robust, further research is still needed for verification and refinement. Future studies should focus on longitudinal measurements of lifestyle factors to provide more comprehensive evidence. For example, obesity is a common influencing factor of GERD. If weight loss is adopted as a new healthy lifestyle, can it promote the reduction of GERD occurrence? We did not conduct relevant data analysis in this article. In addition, mechanistic studies are also needed to elucidate the biological pathways between lifestyle behaviors and GERD risk.

These findings have important implications for public health, underscoring the need for integrated, multilevel interventions to promote healthy lifestyles. By addressing modifiable risk factors at the individual, community, and societal levels, we can mitigate the burden of GERD and improve the quality of life for millions of individuals worldwide.

## 5 Conclusions

The data and analysis from this study provide compelling evidence that engaging in more healthy lifestyle behaviors, namely complete smoking cessation, maintaining optimal sleep, engaging in regular high-intensity exercise, and moderate alcohol consumption, are strongly associated with a significantly reduced risk of developing GERD later on. These findings have profound implications for public health, as they clearly indicate that lifestyle modification should be prioritized and regarded as a key strategy for preventing GERD.

## CRediT authorship contribution statement

**Zhiyu Wang:** Writing – original draft, Investigation, Formal analysis, Conceptualization. **Siwei Lin:** Writing – original draft, Validation, Formal analysis. **Hanwen Liu:** Writing – review & editing, Methodology, Data curation, Conceptualization. **Jianlong Zhou:** Writing – original draft, Software, Methodology, Conceptualization. **Enpei Tang:** Resources, Methodology, Formal analysis. **Zhihui Xi:** Writing – review & editing, Supervision, Software, Resources. **Jiabin Zheng:** Writing – review & editing, Software, Resources. **Wei Yang:** Supervision, Project administration, Funding acquisition.

## Ethics approval and consent to participate

The UK Biobank study was approved by the North West Multi-Center Research Ethics Committee, the National Information Governance Board for Health and Social Care in England and Wales, and the Community Health Index Advisory Group in Scotland. All participants provided written informed consent.

## Funding

We acknowledge financial support from Key-Area 10.13039/100006190Research and Development Program of Guangdong Province (2023B1111020008), and 10.13039/100005930Guangdong Basic and Applied Basic Research Foundation (2024A1515010664), and WU JIEPING MEDICAL FOUNDATION (320.6750.2025-18-88).

## Declaration of competing interest

The authors declare that they have no known competing financial interests or personal relationships that could have appeared to influence the work reported in this paper.

## Data Availability

Due to privacy and sensitive information, the data from this study will not be made public. UK Biobank data is available to approved researchers (https://www.ukbiobank.ac.uk/). Relevant data can be obtained upon reasonable request to the corresponding authors.

## Appendix A Supplementary data

Supplementary material 1 Details about the ascertainment of exposure and outcome Supplementary material 1

Supplementary material 2 Risk functions of Gastroesophageal reflux disease in relation to follow-up time Supplementary material 2

Supplementary material 3 Associations between healthy lifestyle behaviors (participants who did not consume any alcohol were considered to have a healthy alcohol drinking behavior) and the risk of incident Gastroesophageal reflux disease* Supplementary material 3

Supplementary material 4 Associations between healthy lifestyle behaviors (participants who did not diagnose with Gastroesophageal reflux disease in the first two years following baseline) and the risk of incident Gastroesophageal reflux disease* Supplementary material 4

Supplementary material 5 Associations between healthy lifestyle behaviors (Educational attainment and race are also included as confounding factors) and the risk of incident Gastroesophageal reflux disease* Supplementary material 5
