# Supplementary material for: Association of healthy lifestyle behaviors with incident gastroesophageal reflux disease in a large population-based prospective cohort
Source: Prev Med Rep. 2025 Oct 24;60:103276. doi: 10.1016/j.pmedr.2025.103276 (PMC12666430; doi:10.1016/j.pmedr.2025.103276)
Supplement: Supplementary material 1 — Details about the ascertainment of exposure and outcome [file mmc1.docx]

**Text S1 Details about the ascertainment of exposure and outcome**

According to the past and current smoking status reported at baseline, the participants were grouped into corresponding categories of smoking (previous, current, never, or prefer not to answer). Only participants who never smoked were considered healthy in this aspect.

Physical activity of different intensities (walking, moderate, and vigorous) in a typical week was measured in metabolic equivalent task-hour (MET-h) at baseline using the International Physical Activity Questionnaire.^1^ Examples of moderate physical activity include carrying light loads or cycling at a normal pace. Vigorous physical activity is defined as activity that makes one sweat and breathe hard, such as fast cycling, aerobic exercise, and heavy lifting. With reference to a previous study, participants who performed vigorous physical activity in the highest 50% of the cohort were defined as having a healthy level of physical activity.^2^

The quality of sleep was determined based on self-reported sleep duration and sleep problems at baseline. According to previous studies and recommendations on sleep, participants who reported sleeping 7 to 9 h/day and who were without any of the following three sleep problems were considered to have optimal sleep: insomnia (answered ‘never or rarely’ for the question ‘Do you have trouble falling asleep at night or do you wake up in the middle of the night?’), narcolepsy (answered ‘never or rarely’ for the question ‘How likely are you to doze off or fall asleep during the daytime when you don’t mean to?’), and difficulty getting up in the morning (answered ‘fairly easy or very easy’ for the question ‘On an average day, how easy do you find getting up in the morning?’).^3-4^

Diet was estimated by five rounds of a Web-based 24-hour dietary recall questionnaire, the Oxford WebQ.^5^ The first round was conducted in one of the UK Biobank assessment centers at baseline during 2009 to 2010, and the subsequent four rounds were completed online between 2011 and 2012 at three- to four-month intervals. The questionnaire asked if the participants had consumed any of 206 types of food or 32 types of beverages during the preceding 24 hours.^6^ Positive answers would lead to further questions about the specific type of food and the standard serving categories or portion sizes consumed. We tried to capture participants’ habitual diets, so only those who completed at least two rounds of the dietary questionnaire were included, and their intakes were averaged for all available questionnaires. In addition, the UK Biobank provided estimates of nutrient intake, including sodium and alcohol, based on the participants’ answers to the dietary questionnaire. Details about the nutrient calculations have been described elsewhere. Dietary quality was evaluated based on adherence to the Dietary Approaches to Stop Hypertension (DASH) diet.^7^ DASH consists of eight food components: fruits, vegetables, nuts and legumes, low-fat dairy products, whole grains, sodium, red and processed meats, and sweetened beverages. According to their DASH diet scores, calculated based on their consumption of each of the eight food components, the participants were classified into quintiles. Because higher intake of the first five foods was desired, their consumption was rewarded (i.e., the participants in the highest quintile received 5 points, whereas those in the lowest, 2nd, 3rd, and 4th quintiles received 1, 2, 3, and 4 points, respectively). Conversely, the intake of the latter three food components was discouraged. Therefore, the participants in the highest quintile received 1 point, whereas those in the lowest quintile received 5 points. Because of the high proportion of participants who did not consume nuts and legumes or sweetened beverages, quartile ranking was used rather than quintile ranking to classify the participants based on the consumption of these two food components. However, the rewarding rationale was the same as above. The eight food component scores were then added up to derive an overall DASH diet score ranging from 5 to 38. Participants in the highest quartile of the DASH diet score were considered to have high dietary quality.^8^

Some longitudinal studies have suggested that moderate alcohol consumption (e.g. between 5 and 15 g/day) is associated with a lower risk of all-cause mortality than abstaining and binge drinking. Additionally, a cross-sectional study reported that alcohol consumption has protective effects in reducing the risk of IBS. Hence, moderate alcohol intake between 5 and 15 g/day was defined as healthy alcohol consumption behavior in this study.^9^

The outcome of interest of the study was the incidence of GERD, which was defined as a new diagnosis under the International Classification of Diseases, 10th Revision (ICD-10) code K21 after baseline assessment. The UK Biobank has summarized the dates of the first occurrences of a range of health-related outcomes mapped to ICD10 codes from various sources, including primary care data, hospital inpatient data, death register records, and self-reported medical conditions, which are updated on a regular basis. The UK Biobank has been liaising with various computer system suppliers (TPP and Vision Health in England, EMIS Health and Vision in Wales, and EMIS Health and Vision Health in Scotland) to obtain the primary care data of participants who have provided written consent for linking their health-related records to the UK Biobank. Hospital inpatient information is from various datasets across the UK: Hospital Episode Statistics Admitted Patient Care (England), Patient Episode Database for Wales Admitted Patient Care (Wales), and General/Acute Inpatient and Day Case-Scottish Morbidity Record (Scotland). The death register records are provided by National Health Service (NHS) Digital for participants in England and Wales and by NHS Central Register for those in Scotland. Self-reported medical conditions were collected at baseline and three repeat assessment visits at a UK Biobank assessment center. Based on the information provided by these sources, the date when the diagnosis of IBS was first made could be identified.

Reference

1. Craig CL, Marshall AL, Sjöström M, et al. International physical activity questionnaire: 12-country reliability and validity. Medicine & science in sports & exercise 2003;35(8):1381-95.

2. Yévenes-Briones H, Caballero FF, Banegas JR, et al. Association of Lifestyle Behaviors With Hearing Loss: The UK Biobank Cohort Study. Mayo Clinic Proceedings 2022;97(11):2040-49. doi: 10.1016/j.mayocp.2022.03.029

3. Fan M, Sun D, Zhou T, et al. Sleep patterns, genetic susceptibility, and incident cardiovascular disease: a prospective study of 385 292 UK biobank participants. European heart journal 2020;41(11):1182-89.

4. Panel CC. Recommended Amount of Sleep for a Healthy Adult: A Joint Consensus Statement of the American Academy of Sleep Medicine and Sleep Research Society. Sleep 2015;38(6):843-44. doi: 10.5665/sleep.4716

5. Liu B, Young H, Crowe FL, et al. Development and evaluation of the Oxford WebQ, a low-cost, web-based method for assessment of previous 24 h dietary intakes in large-scale prospective studies. Public health nutrition 2011;14(11):1998-2005.

6. Perez-Cornago A, Pollard Z, Young H, et al. Description of the updated nutrition calculation of the Oxford WebQ questionnaire and comparison with the previous version among 207,144 participants in UK Biobank. European journal of nutrition 2021;60(7):4019-30.

7. Sacks FM, Svetkey LP, Vollmer WM, et al. Effects on blood pressure of reduced dietary sodium and the Dietary Approaches to Stop Hypertension (DASH) diet. New England journal of medicine 2001;344(1):3-10.

8. Fung TT, Chiuve SE, McCullough ML, et al. Adherence to a DASH-style diet and risk of coronary heart disease and stroke in women. Archives of internal medicine 2008;168(7):713-20.

9. Mayer-Davis E, Leidy H, Mattes R, et al. Alcohol Consumption and All-Cause Mortality: A Systematic Review. 2020
