# Supplementary material for: Association of healthy lifestyle behaviors with incident gastroesophageal reflux disease in a large population-based prospective cohort
Source: Prev Med Rep. 2025 Oct 24;60:103276. doi: 10.1016/j.pmedr.2025.103276 (PMC12666430; doi:10.1016/j.pmedr.2025.103276)
Supplement: Supplementary material 2 — Risk functions of Gastroesophageal reflux disease in relation to follow-up time [file mmc2.docx]

**Fig. S1. Risk functions of Gastroesophageal reflux disease in relation to follow-up time**

**
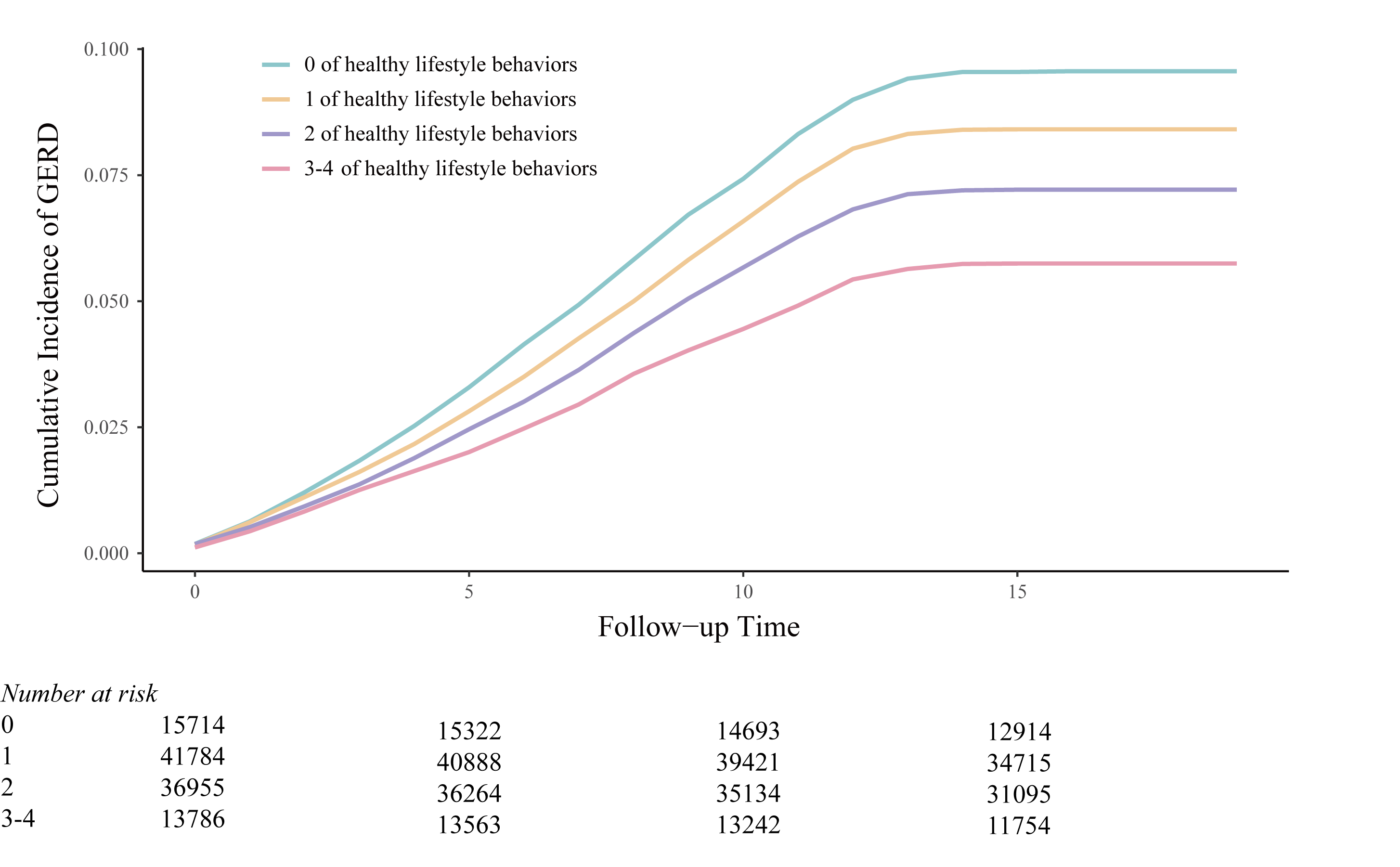
**

GERD, Gastroesophageal reflux disease

Healthy lifestyle behaviors included never smoking, a high level of vigorous physical activity (in the highest 50% of the cohort), moderate alcohol intake (5–15 g/day) and optimal sleep (having a sleep duration between 7 and 9 h/day, finding it fairly easy or very easy to get up in the morning, and never or rarely having insomnia and narcolepsy).The follow-up time is defined as the duration starting from the time of the first blood draw until the diagnosis of gastroesophageal reflux disease, at which point the follow-up concludes.
