# Supplementary material for: Association of healthy lifestyle behaviors with incident gastroesophageal reflux disease in a large population-based prospective cohort
Source: Prev Med Rep. 2025 Oct 24;60:103276. doi: 10.1016/j.pmedr.2025.103276 (PMC12666430; doi:10.1016/j.pmedr.2025.103276)
Supplement: Supplementary material 5 — Associations between healthy lifestyle behaviors (Educational attainment and race are also included as confounding factors) and the risk of incident Gastroesophageal reflux disease* [file mmc5.docx]

**Table S3**. **Associations between healthy lifestyle behaviors (Educational attainment and race are also included as confounding factors) and the risk of incident Gastroesophageal reflux disease***

|  | Number of healthy lifestyle behaviors ^a^ | | | | |
| --- | --- | --- | --- | --- | --- |
|  | 0 | 1 | 2 | 3-4 | P value for trend |
| No. of participants | 15714 (14.5) | 41784 (38.6) | 36955 (34.1) | 13786 (12.7) |  |
| Person years | 89500.0 | 224666.7 | 151235.3 | 51333.3 |  |
| No. of GERD events | 1432 (9.1) | 3370 (8.1) | 2571 (7.0) | 770 (5.6) |  |
| Adjusted hazard ratio ^b^ (95%CI) | 1.00(reference) | 0.93 (0.87 to 0.99) | 0.84 (0.78 to 0.89) | 0.70 (0.64 to 0.77) | <0.01 |

GERD, Gastroesophageal reflux disease

*Values are numbers (percentages) unless stated otherwise

^a^ Healthy lifestyle behaviors included never smoking, a high level of vigorous physical activity (in the highest 50% of the cohort), zero alcohol intake, and optimal sleep (having a sleep duration of between 7 and 9 h/day, finding it fairly easy or very easy to get up in the morning and never or rarely having insomnia and narcolepsy).

^b^ Hazard ratios were adjusted for age (<50, 50–59 or >=60 years), sex (female or male), BMI (<25 or >=25), depression (yes or no), education (yes or no) and ethnicity (yes or no).
